# Clinical Application of Anomalous Muscles for Tendon Transfer in the Upper Limb: A Systematic Review of the Literature

**DOI:** 10.7759/cureus.7222

**Published:** 2020-03-09

**Authors:** Sreenivasulu Metikala, Riaz Mohammed, Venkata Ramana Vakamallu

**Affiliations:** 1 Orthopaedics, Penn State Milton S. Hershey Medical Center, State College, USA; 2 Orthopaedics, University of Pennsylvania Health System, Philadelphia, USA; 3 Orthopaedics, Salford Royal NHS Foundation Trust, Salford, GBR; 4 Orthopaedics, Royal Derby Hospital, Derby, GBR

**Keywords:** accessory muscle, anomalous muscle, upper extremity, tendon transfer

## Abstract

Numerous anomalous muscles of the forearm and hand have been reported in the literature. The majority were encountered in cadaver dissections and some were incidentally detected during tendon transfer surgery. Because of the limited number of motors available for transfer, it may be advantageous if an anomalous muscle with favorable anatomy can function as a potential donor in a suitable clinical environment. Although several authors have illustrated various anomalies and their functional significance, the reports of actual utilization of such muscles as donors for tendon reconstructions are sparse. The aim of the study is to conduct a systematic review of the clinical applications of anomalous muscles in the upper extremity. After a thorough search of PubMed, Web of Science, Scopus, and Cochrane Library databases, only three out of 106 studies were found to be relevant. Two of them discussed the anomalous radial wrist extensor tendon transfer for thumb flexion. The third study described the usage of anomalous flexor carpi ulnaris (FCU) for thumb opposition and index finger flexion, and also proposed a classification. This is the first systematic review of the clinical application of anomalous muscles as donors in the upper extremity tendon transfer surgical procedures. Knowledge of the above classification helps in intraoperative evaluation of the type of the anomaly and the possible consideration of anomalous muscle as a source of transplant material in an appropriate clinical setting.

## Introduction and background

A tendon transfer is a routine procedure in the upper extremity performed to restore the lost function. It is not uncommon to encounter an anomalous or supernumerary muscle in the operating field. This is a surgical challenge as, currently, there are no clear guidelines on how to proceed in such situations. As tiny incisions are practiced routinely during such procedures, these anomalies can obscure the anatomy [1]. Further, it can be confusing to select the donor between the native and anomalous tendons. Numerous authors have recognized various muscular anomalies and incidence and respective patterns in the upper extremity. Most of the studies were either about anatomical findings in cadavers or those found incidentally in the operating field [2-11]. Although the majority of anomalous muscles are asymptomatic, they may occasionally produce persistent pain, unexplained mass, or compression neuropathy necessitating surgical excision [7,8,11-17]. Despite several anatomical descriptions, the actual reports of the clinical application of such anomalous muscles are sparse. Because of the limited number of motors available for transfer, an anomalous muscle with favorable anatomy may function as a potential donor in a suitable clinical environment. It has an additional advantage of preventing residual functional deficit as the native muscle may often be left undisturbed.

The aim of the study is to review the literature for reported cases of anomalous muscles being utilized for tendon transfer procedures in the upper extremity. Every emphasis has been laid to detect and analyze the source muscle, type of anomaly, end result with possible donor-site deficits, and any proposed guidelines for decision-making.

## Review

Materials and methods

Literature Search

According to the Preferred Reporting Items for Systematic Reviews and Meta-Analyses (PRISMA) guidelines, we conducted a comprehensive electronic search using PubMed, Web of Science, Scopus, and Cochrane Library databases on January 19, 2020. The general search terms used were “accessory muscle or anomalous muscle or supernumerary muscle” and “tendon transfer or tendon transplant.” All English-language articles that described the clinical application of anomalous muscles in the upper extremity were reviewed with no date restriction. The lower extremity-related articles were excluded.

Data Extraction

Two authors (SM and RM) independently screened the titles and abstracts of all relevant publications using the above search string. It was combined with an additional manual search of popular journals in the field. Reference lists from the selected articles and bibliographies were also explored for additional articles. The third author (VV) served as a tie-breaker for discrepancies. The relevant studies were further scrutinized by going through the respective full-text articles. Every emphasis was laid to review the source muscle, type of anomaly, the end result with possible donor-site deficits, and any proposed guidelines for decision-making.

Results

**Figure 1:**
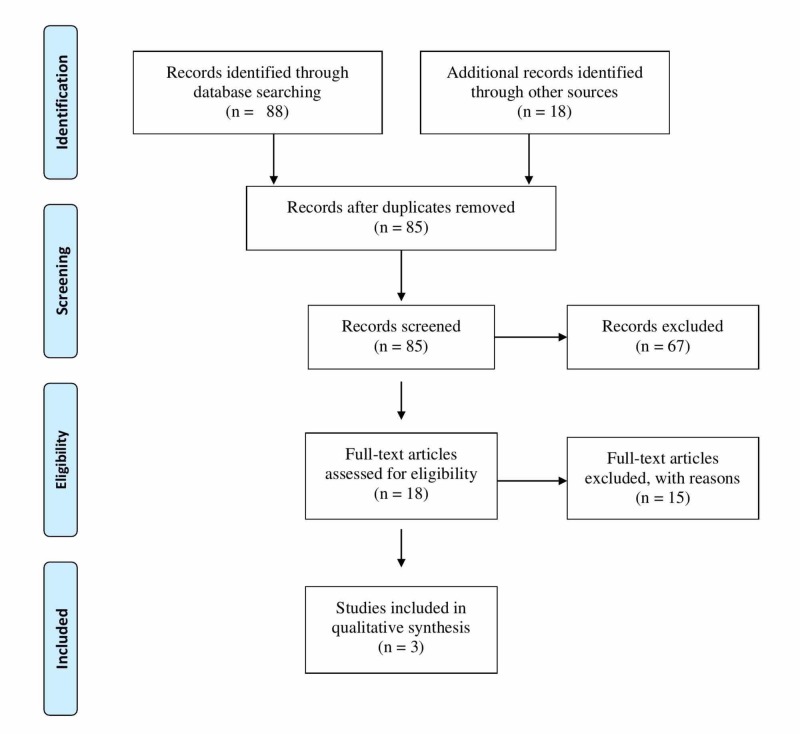
Flow diagram of the systematic review

A total of 106 studies were identified in the initial literature search: 56 in PubMed, 15 in Web of Science, 14 in Scopus, three in the Cochrane Library; the remaining 18 were located through other sources. A flow diagram of our search process is featured in Figure 1.

After removing 21 duplicate studies, 85 studies were kept for further analysis. Of these, 67 were excluded as their titles and abstracts did not meet the inclusion criteria. The full-text articles of the remaining 18 were thoroughly scrutinized for the details, of which only three articles were finalized to illustrate the clinical application of anomalous muscles. A detailed description of these three studies is provided in Table 1.

**Table 1:** Studies analyzing the clinical applications of anomalous muscles in upper extremity ECRI: extensor carpi radialis intermedius; FCU: flexor carpi ulnaris; FPL: flexor pollicis longus; EPB: extensor pollicis brevis; EPL: extensor pollicis longus; FDP: flexor digitorum profundus; APB: abductor pollicis brevis

Author	Year	Patients	Indications	Tendon transfer		Outcomes		Comments	
Zancolli [18]	1975	76	Traumatic quadriplegia	ECRI to FPL		Not specified		No mention on how many of 76 had the transfer	
										Albright and Linburg [19]	1978	3	2: traumatic quadriplegia	ECRI to EPB		1: no follow-up; 1: poor function			
1: radial nerve laceration	ECRI to EPL		Excellent function																
					Bhardwaj et al. [20]	2013	1	Post-Volkmann's ischaemic contracture deficits	Digastric FCU to FDP (index finger) and APB (thumb)				Independent movement of both digits and reasonable hand function at 1-year follow-up		Proposed classification for anomalous FCU				

Due to the limited number of studies and heterogeneity, a meta-analysis was deemed inappropriate, and a narrative approach was used.

The first study was by Zancolli in 1975, who transferred the anomalous wrist extensor, extensor carpi radialis intermedius (ECRI) to flexor pollicis longus (FPL) in quadriplegic hands with preserved active wrist extension [18]. He termed ECRI as the “intermediate radial muscle of Wood,” after the surgeon who had originally described this muscle. ECRI is usually located between extensor carpi radialis longus (ECRL) and extensor carpi radialis brevis (ECRB). Zancolli studied 97 patients with complete traumatic quadriplegia and performed a two-stage reconstruction on 76 patients with multiple tendon transfers and fusion of carpometacarpal joint of the thumb. He found that 68.4% of patients had anomalous wrist extensor tendons either in the form of ECRI muscle or multiple accessory tendinous bands. He transferred ECRI, whenever available, to FPL to obtain an independent flexion of thumb for the lateral pinch. However, there was no mention of how many of 76 actually had ECRI and also no postoperative follow-up was specified. Subsequently, Albright and Linburg, in 1978, presented three cases of anomalous muscle transfer using the same ECRI [19]. The first patient was a 19-year-old male with C5-6 traumatic quadriplegia, who received the transfer of ECRI to extensor pollicis brevis (EPB) in conjunction with other tendon transfer procedures. However, no follow-up was shown. The second patient was a 26-year-old man with an irreparable radial nerve laceration in the proximal forearm. He was treated by ECRI transfer to extensor pollicis longus (EPL) with an excellent function at five years following the operation. In the third case, which was of a 20-year-old woman with C5-6 quadriplegia, the transferred ECRI muscle did not yield functional outcome as it was tiny and insufficient.

The last description of the practical utilization of anomalous muscle in upper extremity was published by Bhardwaj et al. in 2013 [20]. They reported a 31-year-old male patient, with the status of post-complex post-Volkmann’s ischemic contracture deficits with multiple previous surgeries, being planned for a tendon transfer to improve thumb opposition and index finger flexion. Intraoperatively, the authors noted an anomalous flexor carpi ulnaris (FCU) with two independent muscle heads and corresponding muscular bellies. Both bellies had their own tendons, which remained separate until their insertion with independent mobility. The authors termed it a “digastric FCU” and transferred one of the two tendons to the flexor digitorum profundus (FDP) of the index finger. The other tendon was augmented with a free palmaris longus graft and inserted to abductor pollicis brevis (APB) for thumb opposition. Postoperatively, the patient had independent movements of both digits and also attained a reasonable functional outcome at the one-year follow-up. The authors also proposed a new classification for anomalous FCUs into three types based on the clinical appearance and the embryological basis of the anomalies.

Discussion

Anomalies of flexor and extensor tendons are relatively common in the upper extremity. They have been widely reported in the literature with various terms such as anomalous muscle, supernumerary muscle, or accessory muscle [3,4,6,21,22]. It is not uncommon to encounter such a muscle incidentally during a routine tendon transfer surgery, which presents a challenging situation for the surgeon to decide on how to proceed. It may be advantageous if the anomalous tissue can be employed for the desired outcome by sparing the native tendons to avoid donor-site disability. Our study has reviewed the literature on the practical applications of such anomalous muscles in the upper extremity and for any proposed guidelines. Multiple studies of anomalous muscles were identified in the search that discussed their functional significance. For example, Wood, after exploring 312 arms from 156 cadavers in 1988, recognized that 12% had a functional transferable ECRI [23]. Further, 111 out of 312 arms had at least one, often several, accessory tendinous slips at the extensor aspect of the wrist that would be suitable for transfer. In 2008, Nayak et al., in an anatomical study, observed that five out of 48 upper limbs (10.41%) had anomalous muscles of radial wrist extensors [22]. After conducting morphometric measurements, they proposed that these anomalous muscles may help in tendon transfer surgeries. Alshamam et al. depicted two cases of V-shaped bifid palmaris longus muscle in 2010 and believed that they may be used in tendon transfer [9]. Rao et al. in 2011 endorsed the idea of an anomalous flexor digitorum superficialis being a donor for possible tendon transfer [1].

While the above authors illustrated various anomalous muscles and their functional significance, the studies of actual clinical application of such muscles as donors for tendon reconstructions are limited. Our systematic review could identify only three such studies: Zancolli in 1975, Albright and Linburg in 1978, and Bhardwaj et al. in 2013. Although Zancolli called attention to the possibility of using anomalous muscles as transfers in 1968, the surgical reconstruction details were only elaborated in his later study of 96 cases, published in 1975 [18,24]. He performed the transfer of ECRI, whenever available, to FPL as a part of two-stage reconstruction to improve function in quadriplegic hands with preserved active wrist extension. However, no followup was stated in any of the cases. Albright and Linburg shared their experience of ECRI transfer on three patients and noted poor strength in one patient who had received tendon transfer with a tiny ECRI, highlighting that the anatomy of anomaly is an important consideration before its utilization for transfer [19]. Bhardwaj et al. made a significant contribution in 2013 when they described the successful application of an anomalous FCU, and also proposed a novel classification that offered guidelines for intraoperative decision-making [20]. The classification summarized all the reported cases of supernumerary FCUs in the literature into three major groups of anomalies based on the clinical appearance and the embryological basis. Type 1 is a single muscle with split tendon, where the FCU tendon splits before insertion. Type 2 is a digastric FCU with each head forming separate muscle and tendon. Importantly, both heads are innervated independently by the ulnar nerve. Type 3 is an accessory FCU, an extra muscle with features of naïve FCU. The above classification also included the associated anomalies and innervations pattern.

We realize that the relevance of Bhardwaj et al.'s classification goes beyond the description of gross anatomy and can be applied to any anomalous muscle that is intended for possible transfer. As the presence of a functional nerve-muscle-tendon unit is a dominant factor for a functional outcome, we believe that Bhardwaj et al.'s Type 2 has the most appropriate anatomy and either of the two bellies can be chosen for tendon transfer. If Type 1 anomaly is encountered, we recommend the use of both tendons as the donor for transfer. The use of only one tendon can produce a difference in tension between the two tendons, akin to the quadriga effect. In the case of Type 3, as the anomalous muscle does not represent an independent neuro-muscular unit, we prefer the native muscle to function as a donor for all practical applications. Kunc et al., in 2019, further divided the Type 3 into subtypes A and B based on the accessory muscle insertion [25]. Type 3A has insertion to pisiform, triquetral, and abductor digiti minimi, similar to naïve FCU, whereas 3B is attached to the flexor retinaculum and palmar aponeurosis just like palmaris longus. Earlier authors expressed concerns about the practice of anomalous muscle for tendon transfer due to the challenges in assessing its morphology and fulfilling its role as a successful donor [1]. This is where we believe Bhardwaj et al.'s classification is aptly suited for decision-making. By simply extending the incision, the surgeon can make a quick assessment of the type of the anomaly and proceed further as per the above guidelines.

To our best knowledge, this is the first systematic review discussing the muscle anomalies as potential donors for tendon transfer procedures in the upper extremity. We also present evidence that supports their practical application based on anatomical and clinical standpoints. Awareness of this information will help the surgeon for better orientation intraoperatively when faced with incidental anomalous structures. Further, the proposed guidelines based on Bhardwaj et al.'s classification will assist in the decision-making in an appropriate clinical setting that requires tendon transfer. Our study is not without its drawbacks. The most significant drawback is the limited number of clinical applications. However, combined with the available anatomical and clinical studies, we believe there is enough merit to support our recommendations. Finally, with the advent of the “wide-awake local anaesthesia no tourniquet” (WALANT) technique in hand and upper extremity surgery, it is now possible to intraoperatively scrutinize the anomalous muscle by an active motion by the patient [26]. The patient's ability to move the muscle voluntarily and independently makes it a suitable donor for tendon transfer. Also, the amount of tension and excursion for tendon transfers can be adjusted in addition to checking the integrity of repair before wound closure, thus making WALANT an important tool in the surgeon's armamentarium.

## Conclusions

Anomalous muscles can be clinically utilized for tendon transfer without any donor deficit. The current study is the first systematic review conducted on the clinical utilization of anomalous muscles in tendon transfer procedures. In this review, we report both anatomical studies and clinical applications supporting this idea. Different anomalous muscles have been used for tendon transfer although the overall cases are limited. Based on this review, when encountered with an anomaly intraoperatively, we recommend extending the incision to identify the type of anomaly. An anomaly with a separate musculotendinous unit is most suitable for tendon transfer.
